# Identifying the Reactive Metabolites of Tyrosine Kinase Inhibitor Pexidartinib In Vitro Using LC–MS-Based Metabolomic Approaches

**DOI:** 10.1021/acs.chemrestox.3c00164

**Published:** 2023-08-02

**Authors:** Xuan Qin, Yong Wang, Kevin R. MacKenzie, John M. Hakenjos, Si Chen, Saleh M. Khalil, Sung Yun Jung, Damian W. Young, Lei Guo, Feng Li

**Affiliations:** † Center for Drug Discovery, Department of Pathology & Immunology, Baylor College of Medicine, Houston, Texas 77030, United States; ‡ NMR and Drug Metabolism Core, Advanced Technology Cores, Baylor College of Medicine, Houston, Texas 77030, United States; § Department of Pharmacology & Chemical Biology, Baylor College of Medicine, Houston, Texas 77030, United States; ∥ Division of Biochemical Toxicology, National Center for Toxicological Research/U.S. Food and Drug Administration (FDA), Jefferson, Arkansas 72079, United States; ⊥ Department of Molecular & Cellular Biology, Baylor College of Medicine, Houston, Texas 77030, United States

## Abstract

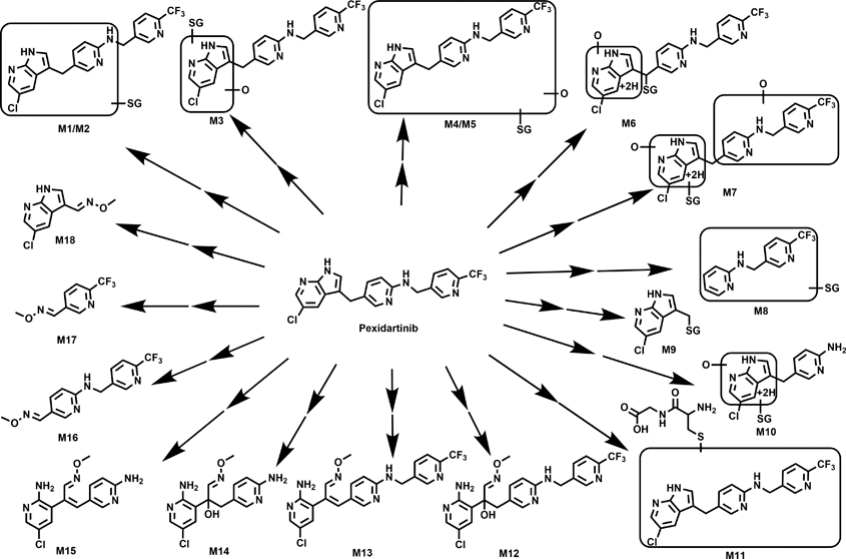

Pexidartinib (PEX, TURALIO), a selective and potent inhibitor of the macrophage colony-stimulating factor-1 receptor, has been approved for the treatment of tenosynovial giant cell tumor. However, frequent and severe adverse effects have been reported in the clinic, resulting in a boxed warning on PEX for its risk of liver injury. The mechanisms underlying PEX-related hepatotoxicity, particularly metabolism-related toxicity, remain unknown. In the current study, the metabolic activation of PEX was investigated in human/mouse liver microsomes (HLM/MLM) and primary human hepatocytes (PHH) using glutathione (GSH) and methoxyamine (NH_2_OMe) as trapping reagents. A total of 11 PEX-GSH and 7 PEX-NH_2_OMe adducts were identified in HLM/MLM using an LC–MS-based metabolomics approach. Additionally, 4 PEX-GSH adducts were detected in the PHH. CYP3A4 and CYP3A5 were identified as the primary enzymes responsible for the formation of these adducts using recombinant human P450s and CYP3A chemical inhibitor ketoconazole. Overall, our studies suggested that PEX metabolism can produce reactive metabolites mediated by CYP3A, and the association of the reactive metabolites with PEX hepatotoxicity needs to be further studied.

## 1 Introduction

Pexidartinib (PEX, TURALIO), a novel small molecule tyrosine kinase inhibitor, has highly selective and potent inhibitory activity against macrophage colony-stimulating factor-1 receptor (CSF1R).^1,2^ It has been approved by the Food and Drug Administration (FDA) for the treatment of tenosynovial giant cell tumor in adults who are not likely to benefit from surgery.^3−6^ PEX is also under investigation for its potential as a monotherapy or combination therapy in various malignancies.^5,7,8^ A recent study indicates that PEX also holds great promise as a treatment for anaplastic thyroid cancer.^9^ Generally, PEX has shown efficacy and tolerability in patients.^7,10^ However, serious adverse reactions caused by PEX were reported in 13% of patients, including life-threatening hepatotoxicity occurring in 3.3% of patients.^4,11^ Due to the potential for severe and even fatal liver injury, the FDA has issued a boxed warning for its hepatotoxicity. PEX is available to patients only through a restricted program under a Risk Evaluation and Mitigation Strategy,^4^ and it is essential to monitor liver function prior to the initiation of PEX treatment.^10,12^

To date, the mechanism(s) of PEX-related toxicity remains mostly unidentified. Drug metabolism is closely connected to both drug efficacy and adverse effects.^13,14^ It is well appreciated that reactive metabolites play a critical role in idiosyncratic adverse drug reactions.^14,15^ According to FDA documents, PEX is rapidly metabolized, primarily by the enzymes CYP3A and UGT1A4, which are involved in phase I and phase II metabolism, respectively.^12^ PEX was recovered in the feces as the unchanged form (44%) and in the urine (27%) as metabolites (≈10% as N-glucuronide).^3,5^ Our previous studies focused on the carbon–carbon bond cleavage in PEX metabolism, provided a comprehensive profile of the phase I metabolism of PEX in human liver microsomes (HLM), and identified its stable metabolites.^16^ However, it is still unclear whether PEX metabolism is capable of generating reactive metabolites and what specific roles they may play in causing its severe hepatotoxicity.

In the present study, we employed LC–MS-based metabolomic approaches to investigate the metabolic bioactivation of PEX in in vitro systems. We used reduced glutathione (GSH) and methoxyamine (NH_2_OMe) as trapping reagents in HLM and mouse liver microsomes (MLM).^17−20^ The drug metabolizing enzymes contributing to the generation of PEX reactive metabolites were determined using recombinant human P450s and a specific chemical inhibitor in HLM/MLM and primary human hepatocytes (PHH). Our studies revealed 11 PEX-GSH and 7 PEX-NH_2_OMe adducts in HLM/MLM. Four PEX-GSH adducts were observed in PHH treated with PEX. These findings from this study may facilitate the understanding of PEX hepatotoxicity from the perspective of drug metabolism.

## 2 Materials and Methods

### 2.1 Materials and Chemicals

PEX, (5-[(5-chloro-1*H*-pyrrolo[2,3-*b*]pyridin-3-yl)methyl]-*N*-[[6-(trifluoromethyl)pyridin-3-yl]methyl]pyridin-2-amine), was purchased from Cayman Chemical (Ann Arbor, MI). Ketoconazole (KCZ), NH_2_OMe hydrochloride, GSH, formic acid, and β-nicotinamide adenine dinucleotide 2′-phosphate reduced tetrasodium salt hydrate (NADPH) were purchased from Sigma-Aldrich (St. Louis, MO). HLM (catalog #: H2630; Lot #: 1910096), MLM (catalog #: M5000; Lot #: 2210070), recombinant human P450s (EasyCYP Bactosomes), and PHH were obtained from XenoTech (Kansas City, KS). All solvents for liquid chromatography and mass spectrometry were of LC–MS grade (Thermo Fisher Scientific, San Jose, CA).

### 2.2 Trapping the Reactive Metabolites of PEX with GSH or NH_2_OMe in Liver Microsomes and Recombinant P450s

Incubations were conducted in 1× phosphate-buffered saline (PBS, pH 7.4) containing 30 μM PEX, 0.1 mg LM (HLM or MLM), or 1 pmol of each cDNA-expressed human P450 enzymes (control, CYP1A2, 2A6, 2B6, 2C8, 2C9, 2C19, 2D6, 2E1, 3A4, and 3A5) and 2.5 mM GSH or 2.5 mM NH_2_OMe in a final volume of 95 μL. After a 5 min preincubation at 37 °C, 5 μL of 20 mM NADPH (final concentration: 1.0 mM) was added to initiate the reactions. The incubation continued for 40 min at 37 °C with gentle shaking. Incubations without NADPH or GSH/NH_2_OMe were used as controls. Co-incubations of PEX (30 μM) and KCZ (human and mouse CYP3A inhibitor, 2 μM) in HLM and MLM with GSH or NH_2_OMe were performed to determine the role of CYP3A in the formation of GSH or NH_2_OMe adducts related to PEX. Reactions were quenched by adding 100 μL of ice-cold acetonitrile and vortexing for 30 s, and the mixtures were then centrifuged at 15,000 rcf for 15 min. The supernatant was transferred to an autosampler vial, and 3 μL was injected into an ultrahigh-performance liquid chromatography (UHPLC) Q Exactive MS system for analysis. Incubations were performed in duplicate for cDNA-expressed P450 enzymes and in triplicate for LM experiments. The PEX concentration (30 μM) used in liver microsomes is clinically relevant according to the *C*_max_ in human subjects.

The average plasma *C*_max_ of PEX is around 4 μg/mL (∼9.6 μM) in human subjects at 400 mg per day.^21^ Typically, the concentration of drugs in the liver is several times greater than that in the bloodstream.

### 2.3 Identifying the Role of CYP3A in the Formation of PEX-GSH Adducts in PHH

PHH (Xenotech, Kansas City, KS, 20 donors, Cat No. HPCH20-50, Batch No. 1910146) were thawed and plated in 12-well plates (Corning, Corning, NY) according to the protocol of the vendor with the density of 6.95 × 10^5^ cells/well. The cells were cultured in complete HepatoZYME medium, containing GlutaMAX, Insulin-Transferrin-Selenium, and penicillin/streptomycin (all of the reagents were obtained from Thermo, San Jose, CA) at 37 °C in a humidified atmosphere with 5% CO_2_ for 24 h before PEX treatment. The PHH were treated with 20 μM PEX with or without 10 μM KCZ for 6 h. The medium was then transferred into Eppendorf tubes and centrifuged at 100 rcf for 5 min to remove the suspending cells. The cells were washed with 1× DPBS (Thermo, San Jose, CA) 3 times, harvested in 500 μL of methanol–water (v/v 1/1), and lysed with a probe ultrasonicator (Thermo, San Jose, CA). Twenty microliters of culture medium was added to 60 μL of ice-cold acetonitrile containing 0.1 μM agomelatine as the internal standard (IS), or 50 μL of cell lysate was added with 100 μL of IS solution. After vortexing and centrifugation at 15,000 rcf for 15 min, the supernatants were transferred to sample vials and 3 μL was injected into a UHPLC-Q Exactive MS system for analysis.

### 2.4 UHPLC-MS Analyses

PEX and its GSH or NH_2_OMe adducts in the samples were resolved, analyzed, and relatively quantified by a UHPLC-Q Exactive MS system (Thermo Fisher Scientific, San Jose, CA). The column for analyte separation was an Acquity 100 mm × 2.1 mm BEH C-18 column (1.7 μm, Waters, Milford, MA), whose temperature was maintained at 40 °C. The flow rate was 0.3 mL/min with gradient ranging from 2 to 95% in the water-acetonitrile mobile phase system both containing 0.1% formic acid in a 15 min run. Q Exactive MS was operated in positive mode with electrospray ionization. Ultrapure nitrogen was applied as the sheath (45 arbitrary unit), auxiliary (10 arbitrary unit), sweep (1.0 arbitrary unit), and the collision gas. The capillary gas temperature was set at 350 °C, and the capillary voltage was set at 4.3 kV. MS data were acquired from 80 to 1200 Da in the profile mode. The ion at *m*/*z* 371.1012 was used as a reference for positive mode during acquisition. The MS/MS of GSH and NH_2_OMe adducts associated with PEX was performed with the normalized collision energy set at 10–35 arbitrary units with an isolation width of 2 *m*/*z*.

### 2.5 Data Analysis

Chromatograms and mass spectra from *m*/*z* 80 to 1200 were acquired in profile format by Xcalibur software (Thermo Fisher Scientific, San Jose, CA). These data were processed by Compound Discoverer 3.1 software (Thermo Fisher Scientific, San Jose, CA) to generate a multivariate data matrix. Data matrices were exported into SIMCA14 (Umetrics, Kinnelon, NJ) for multivariate data analysis. Orthogonal projection to latent structures-discriminant analysis (OPLS-DA) was performed on Pareto-scaled data.^20^ For chemometric analysis, matrix data were processed from *m*/*z* 80 to 1000.

## 3 Results

### 3.1 Profiling PEX-GSH Adducts in Liver Microsomes and PHH

**Figure 1:**
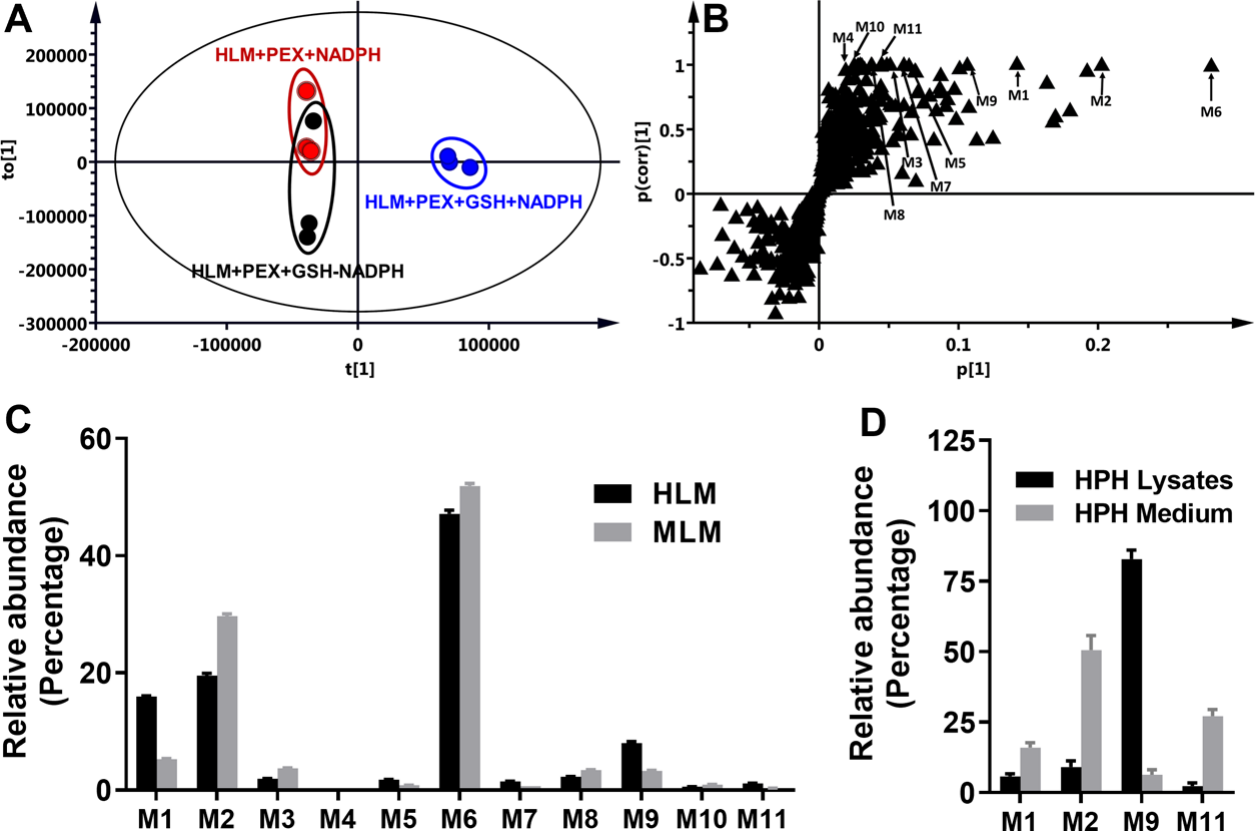
Metabolomic screening of the PEX-GSH adducts in human liver microsomes. Metabolomic analysis was performed on the control groups (HLM + PEX + NAPDH and HLM + PEX + GSH-NAPDH) and the PEX group (HLM + PEX + GSH + NAPDH). The incubations were conducted in 1× PBS (pH 7.4) with a final volume of 100 μL, containing 30 μM PEX, 0.1 mg of LM, 2.5 mM GSH, and NADPH (final concentration, 1.0 mM). The reaction was continued for 40 min with gentle shaking. (A) Separation of control and PEX groups in OPLS-DA score plot. The *t*[1] and *t*[1] values represent the score of each sample in principal components 1 and 2, respectively. (B) Loading S-plot generated by OPLS-DA analysis. The *X*-axis indicates the relative abundance of ions, while the *Y*-axis represents the correlation of each ion to the model. The top-ranking ions associated with PEX-GSH adducts are labeled in the S-plots. The number of ions (metabolite identification) corresponds to those in Table 1. (C) Relative abundance of GSH adducts associated with PEX in HLM and MLM. The relative quantification of trapped metabolites was conducted based on the peak area. The overall abundance of GSH adducts in each sample was set as 100%. The data are expressed as mean ± S.E.M (*n* = 3). (D) Relative abundance of PEX-GSH adducts in PHH lysate and medium. PHH seeded in 12-well plates were treated with 20 μM PEX for 6 h. The incubated samples, PHH medium, and cell lysates samples were all analyzed using the UHPLC-Q Exactive MS system to measure PEX-GSH adducts. The overall abundance of GSH adducts in each sample was set as 100%. The data are expressed as mean ± S.E.M (*n* = 4). PEX, pexidartinib; GSH, reduced glutathione; 1× PBS, 1× phosphate-buffered saline; HLM/MLM, human/mouse liver microsomes; OPLS-DA, orthogonal projection to latent structures-discriminant analysis; PHH, primary human hepatocytes; KCZ, ketoconazole.

The results of the chemometric analysis on the ions produced from the UHPLC-Q Exactive MS analysis of three groups of samples are presented in Figure 1A,B. These groups were generated by incubating PEX in HLM with or without NADPH or GSH. Two of the groups, which did not contain NADPH or GSH, were used as control groups. Principal component analysis conducted on the data revealed three distinguishable clusters, with two clusters corresponding to the control groups and the remaining one associated with the analyte group (as depicted in Figure 1A). These findings suggest differences in the chemical components among the three groups. The S-plot generated by OPLS-DA (Figure 1B) illustrates the ion contributions to the separation of groups in HLM. The top-ranking ions, which were identified as PEX-GSH adducts, were labeled in the S-plot (Figure 1B). The PEX-GSH adducts identified in HLM incubations were comparable to those detected in MLM incubations. Eleven GSH adducts associated with PEX (M1–M11) were determined in LM, and four of them (M1, M2, M9, and M11) were detected in both PHH lysate and medium (Table 1). Their relative abundances in LM and PHH are shown in Figure 1C,D, respectively. In HLM/MLM, M1 (16/5.3%), M2 (19/30%), and M6 (47/52%) were the relatively abundant adducts based on the peak areas (Figure 1C). In PHH lysate, M9 (the GSH adduct of the cleaved left moiety of PEX, Figure S1) was the most prevalent adduct, constituting 83% of the total PEX-GSH adducts. In contrast, M2 was the adduct with the largest peak in the PHH medium representing 51% of the total, while M9 accounted for 6.3% (Figure 1D) based on the peak area.

**Table 1:** Summary of the Adducts Associated with PEX in Liver Microsomes and Human Primary Hepatocytes^a^

RT (min)	observed *m*/*z* [M + H]^+^	calculated *m*/*z* [M + H]^+^	mass error (ppm)	predicted molecular formula	identification	metabolite ID	source
10.96	418.1036	418.1036	0.00	C_20_H_15_ClF_3_N_5_	pexidartinib	PEX	HLM, MLM, PHH
8.86	723.1731	723.1722	1.24	C_30_H_30_ClF_3_N_8_O_6_S	PEX + GSH	M1	HLM, MLM, PHH
8.98	723.1731	723.1722	1.24	C_30_H_30_ClF_3_N_8_O_6_S	PEX + GSH	M2	HLM, MLM, PHH
8.48	739.1682	739.1672	1.35	C_30_H_30_ClF_3_N_8_O_7_S	PEX + O + GSH	M3	HLM, MLM
9.02	739.1677	739.1672	0.68	C_30_H_30_ClF_3_N_8_O_7_S	PEX + O + GSH	M4	HLM, MLM
9.58	739.1678	739.1672	0.81	C_30_H_30_ClF_3_N_8_O_7_S	PEX + O + GSH	M5	HLM, MLM
8.11	741.1839	741.1828	1.48	C_30_H_32_ClF_3_N_8_O_7_S	PEX + O + 2H + GSH	M6	HLM, MLM,
8.64	757.1783	757.1777	0.79	C_30_H_32_ClF_3_N_8_O_8_S	PEX + 2O + 2H + GSH	M7	HLM, MLM
6.27	559.1588	559.1581	1.25	C_22_H_25_ClF_3_N_6_O_6_S	cleaved right + GSH	M8	HLM, MLM
7.51	472.1055	472.1052	0.64	C_18_H_22_ClN_5_O_6_S	cleaved left + GSH	M9	HLM, MLM, PHH
3.88	582.1536	582.1532	0.69	C_23_H_28_ClN_7_O_7_S	amine + O + 2H + GSH	M10	HLM, MLM
8.53	594.1301	594.1296	0.84	C_25_H_24_ClF_3_N_7_O_3_S	PEX + Cys + Gly	M11	HLM, MLM, PHH
9.50	481.1363	481.1361	0.42	C_21_H_20_ClF_3_N_6_O_2_	PEX + O + 2H + NH_2_OMe	M12	HLM, MLM
10.82	463.1265	463.1261	0.86	C_21_H_18_ClF_3_N_6_O	PEX + NH_2_OMe-2H	M13	HLM, MLM
4.74	322.1065	322.1065	0.00	C_14_H_16_ClN_5_O_2_	amine + O + 2H + NH_2_OMe	M14	HLM, MLM
7.09	304.0959	304.0965	–1.97	C_14_H_14_ClN_5_O	amine + NH_2_OMe-2H	M15	HLM, MLM
10.87	311.1113	311.1114	–0.32	C_14_H_13_F_3_N4O	cleaved right + NH_2_OMe^b^	M16	HLM, MLM
12.41	205.0586	205.0583	1.46	C_8_H_7_F_3_N_2_O	nicotinaldehyde + NH_2_OMe	M17	HLM, MLM
12.24	210.0430	210.0429	0.48	C_9_H_8_ClN_3_O	cleaved left + NH_2_OMe^b^	M18	HLM, MLM

a PEX, pexidartinib; GSH, glutathione; NH_2_OMe, methoxyamine; O+, monohydroxylation; 2O+, dihydroxylation; O+2H+, monohydroxylation & hydrogenation; Cys, cysteine; Gly, glycine; HLM/MLM, human/mouse liver microsomes; PHH, primary human hepatocytes; Amine, N-dealkylated PEX.

b See Figure S1 for cleaved moieties.

### 3.2 Identification of PEX-GSH Adducts M1–M11

**Figure 2:**
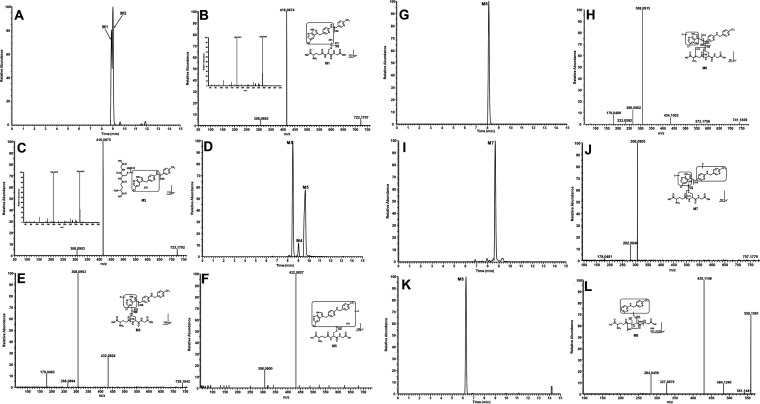
Identification of GSH adducts M1-M8 related to PEX. Incubations and metabolite elucidation conditions in HLM were as described in Figure 1. All of the samples were analyzed using UHPLC-Q Exactive MS. Structural elucidation was performed based on accurate mass (with mass errors less than 5 ppm) and MS/MS fragmentation. MS/MS was performed with collision energy ranging from 10 to 35 eV. For M1 and M2, an additional fragmentation was performed using a higher collision energy of 45 eV. The major fragment ions are interpreted in the insets. (A) Chromatograms of M1 and M2. (B, C) MS/MS of M1 and M2. (D) Chromatograms of M3-M5. (E) MS/MS of M3. (F) MS/MS of M5. (G) Chromatogram of M6. (H) MS/MS of M6. (I) Chromatogram of M7. (J) MS/MS of M7. (K) Chromatogram of M8. (L) MS/MS of M8.

Among 11 GSH adducts associated with PEX, 7 of them were formed by conjugating GSH with PEX (M1 and M2) and oxidized PEX (M3–M7) in the presence of NADPH and GSH in HLM and MLM. Their formation is NADPH- and GSH-dependent, as they were not detected in control groups (representative trend plots of M1 and M8 shown in Figure S2). Protonated molecules with exact masses at *m*/*z* 723.1722 were observed for M1 and M2, eluting at 8.86 and 8.98 min, respectively (Figure 2A). M1 and M2 were identified as the GSH adducts of PEX. At a lower normalized collision energy (30 arbitrary unit), the MS/MS of both M1 and M2 produced two primary fragment ions at *m*/*z* 308.0903 and 416.0875, indicating that GSH was attached to PEX (Figure 2B,C). At a higher normalized collision energy (45 arbitrary unit), fragment ions at *m*/*z* 160.0370 and 255.0435 were generated, as shown in the inlaid MS/MS spectra (Figure 2B,C). The fragment ion at *m*/*z* 160.0370 was also observed in the MS/MS of PEX (Figure S3). Thus, it is likely that GSH in M1 and M2 is not linked to the (6-(trifluoromethyl)pyridin-3-yl)methyl moiety in PEX. The fragment ions were interpreted in the inlaid structural diagrams (Figure 2B,C).

M3-M5 have the same protonated exact mass at *m*/*z* 739.1672 and eluted at 8.48, 9.02, and 9.58 min, accordingly (Figure 2D and Table 1). Their molecular weight is 16 Da higher than those of M1 and M2, which were identified as GSH adducts of monohydroxylated PEX (PEX+O). The MS/MS of M3 produced the fragment ions at *m*/*z* 179.0483, 266.0894, 308.0903, and 432.0824, suggesting GSH is linked to the pyrrolo[2,3-*b*]pyridinyl moiety (Figure 2E). The MS/MS of M5 only generated two fragment ions at *m*/*z* 308.0900 and 432.0827 (Figure 2F). Their fragment ions were interpreted in the inlaid structural diagrams in Figure 2E,F, respectively. Unfortunately, a sufficiently high-quality MS/MS spectrum of M4 could not be obtained. Therefore, M4 was identified as a GSH adduct of PEX+O based on its predicted formula and exact mass (Table 1).

M6 was the GSH adduct with the largest peak in HLM/MLM, eluting at 8.11 min and having a protonated molecule at *m*/*z* 741.1828. It was identified as the GSH adduct of monohydroxylated hydrogenated PEX (PEX+O+2H+GSH) (Figure 2G). The MS/MS spectrum of M6 produced fragmental ions at *m*/*z* 179.0489, 233.0592, 266.0902, 308.0915, 434.1002, and 573.1756. The fragment ions were interpreted in the inlaid structural diagrams in Figure 2H. The presence of fragment ions with *m*/*z* 266.0902 and 573.1756 suggests that GSH is bound to the methylene position. M7, eluting at 8.64 min, exhibited a protonated molecule at *m*/*z* 757.1777, identified as the GSH adduct of dihydroxylated hydrogenated PEX (PEX + 2O + 2H + GSH) (Figure 2I). The MS/MS analysis of M7 showed the presence of fragment ions at *m*/*z* 179.0481, 282.0840, and 308.0915 (Figure 2J), which were interpreted in the inlaid structural diagrams. Compared to the fragment ion at *m*/*z* 266.0894 from M6, the presence of the ion at *m*/*z* 282.0840 in M7 indicated that the second oxidation occurred on the right-framed moiety of PEX.

**Figure 3:**
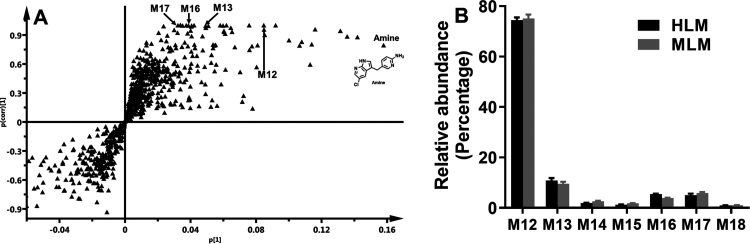
Metabolomic screening of the PEX-NH_2_OMe adduct in human liver microsomes. Metabolomic analysis was performed on control groups (HLM + PEX + NAPDH and HLM + PEX + NH_2_OMe-NAPDH) and the PEX group (HLM + PEX + NH_2_OMe + NAPDH). Incubations were conducted in 1× PBS (pH 7.4) with a final volume of 100 μL, containing 30 μM PEX, 0.1 mg LM, 2.5 mM NH_2_OMe, and NADPH (final concentration 1.0 mM). The reaction continued for 40 min with gentle shaking. (A) Loading S-plot generated by OPLS-DA analysis. The *X*-axis represents the relative abundance of ions, and the *Y*-axis represents the correlation of each ion to the model. The top-ranking ions associated with PEX-NH_2_OMe adducts are labeled in S-plot. The number of ions (metabolite identification) corresponds to those in Table 1. (B) Relative abundance of NH_2_OMe adducts associated with PEX in HLM and MLM. The relative quantification of trapped metabolites was conducted based on the peak area. The overall abundance of NH_2_OMe adducts in each sample was set as 100%. The data are expressed as mean ± S.E.M (*n* = 3).

Interestingly, we also identified three GSH adducts (M8–M10) related to the carbon–carbon cleavage in PEX phase I metabolism, which was investigated in detail in our previous study.^16^ M8 and M9 were generated by conjugating GSH with cleaved parts (Figures 2L and S1). M10 was formed by GSH reacting with the N-dealkylated PEX metabolite (amine, Figures 3A and S4A). M8 eluted at 6.27 min with a protonated *m*/*z* value of 559.1581 (Figure 2K). The MS/MS of M8 produced fragment ions at *m*/*z* 284.0459, 327.0879, 430.1148, 484.1240, and 541.1441, which were interpreted in the inlaid structural diagram (Figure 2L). M9 has a protonated molecule at *m*/*z* 472.1052, and it has been characterized in our previous studies.^16^ The pathway of M9 formation is briefly described in Figure S1. M10, eluted at 3.88 min, has a protonated molecule at *m*/*z* 582.1532 (Figure S4A and Table 1). Additionally, M11, a PEX + cysteine (Cys) + glycine (Gly) adduct, was observed in HLM/MLM and PHH, eluting at 8.53 min with a protonated molecule at *m*/*z* 594.1296 (Figure S4B and Table 1). Unfortunately, we failed to obtain the MS/MS spectra of M10 and M11 with adequate quality for MS/MS analysis. Thus, M10 and M11 were identified as the GSH adduct of amine (amine + O + 2H + GSH) and PEX + Cys + Gly, respectively, based on their exact mass and predicted formulas (Table 1).

### 3.3 Profiling PEX-NH_2_OMe Adducts in Liver Microsomes

Similar to the profiling of PEX-GSH adduct, we performed the chemometric analysis on the ions produced by the UHPLC-Q Exactive MS analysis of three groups of samples. These groups were generated by incubating PEX in HLM with or without NADPH or NH_2_OMe. The principal component analysis resulted in three distinct clusters, as shown in Figure S5A. The S-plot generated from OPLS-DA (Figure 3A) illustrates the ions that contribute to the separation of groups in HLM, including PEX metabolites and PEX-MeONH_2_ adducts, which were marked in the S-plot (Figure 3A). Four NH_2_OMe adducts (M12, M13, M16, and M17) with larger peak areas associated with PEX in LM were determined in LM based on the metabolomic analysis (Table 1). Additionally, we manually identified 3 adducts (M14, M15, and M18) with smaller peak areas. The formation of PEX-NH_2_OMe adducts in HLM followed a similar trend to those in MLM incubations. The relative abundances of these adducts in LM are shown in Figure 3B.

### 3.4 Identification of PEX-NH_2_OMe Adducts M12–M18

**Figure 4:**
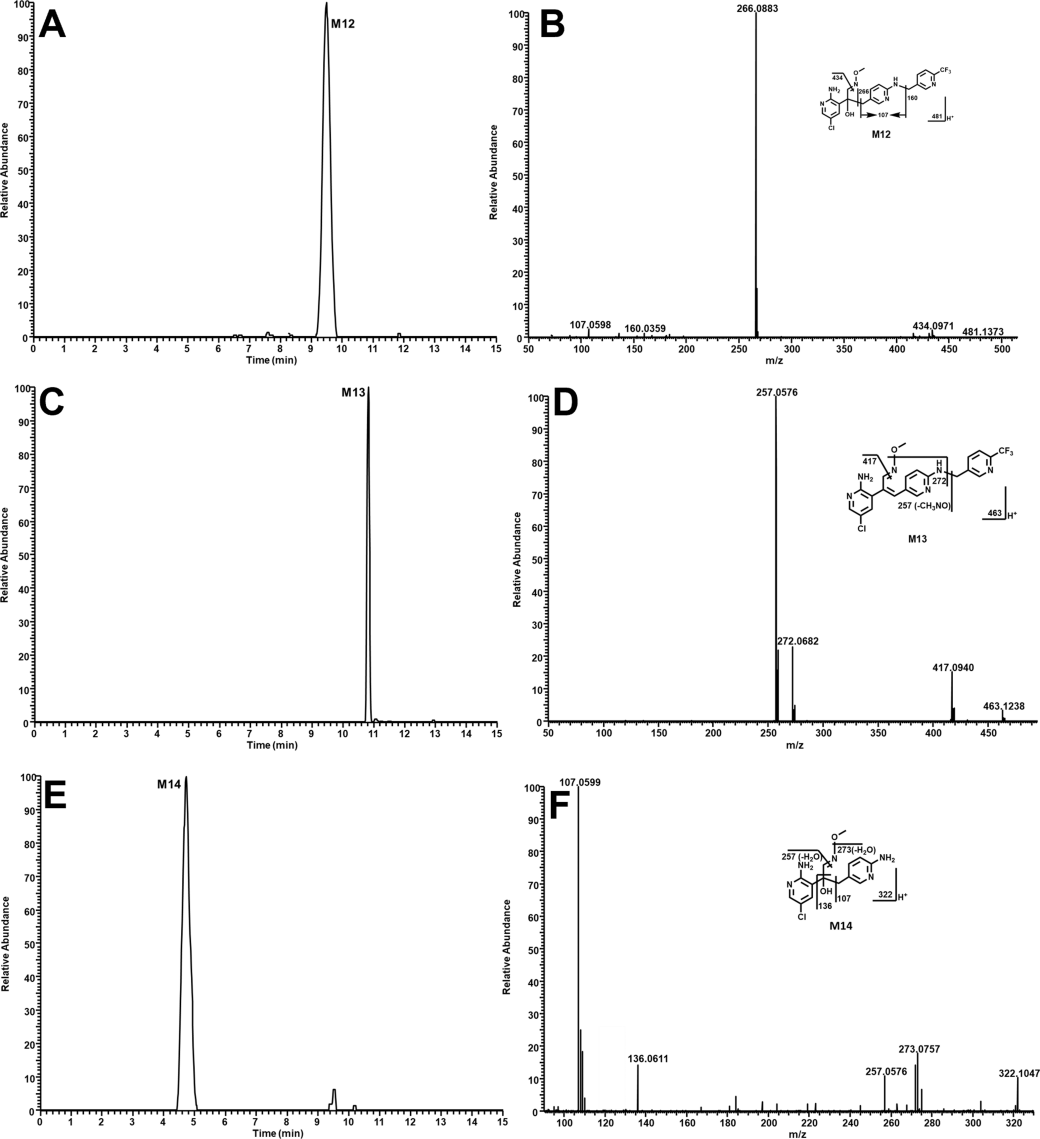
Identifying oximes M12–M14. Incubations and metabolite elucidation conditions in HLM were conducted as described in Figure 3. All of the samples were analyzed using UHPLC-Q Exactive MS. MS/MS was performed with collision energy ramping from 10 to 35 arbitrary unit. (A) Chromatogram of M12 in HLM. (B) MS/MS of M12. (C) Chromatogram of M13. (D) MS/MS of M13. (E) Chromatogram of M14. (F) MS/MS of M15.

**Figure 5:**
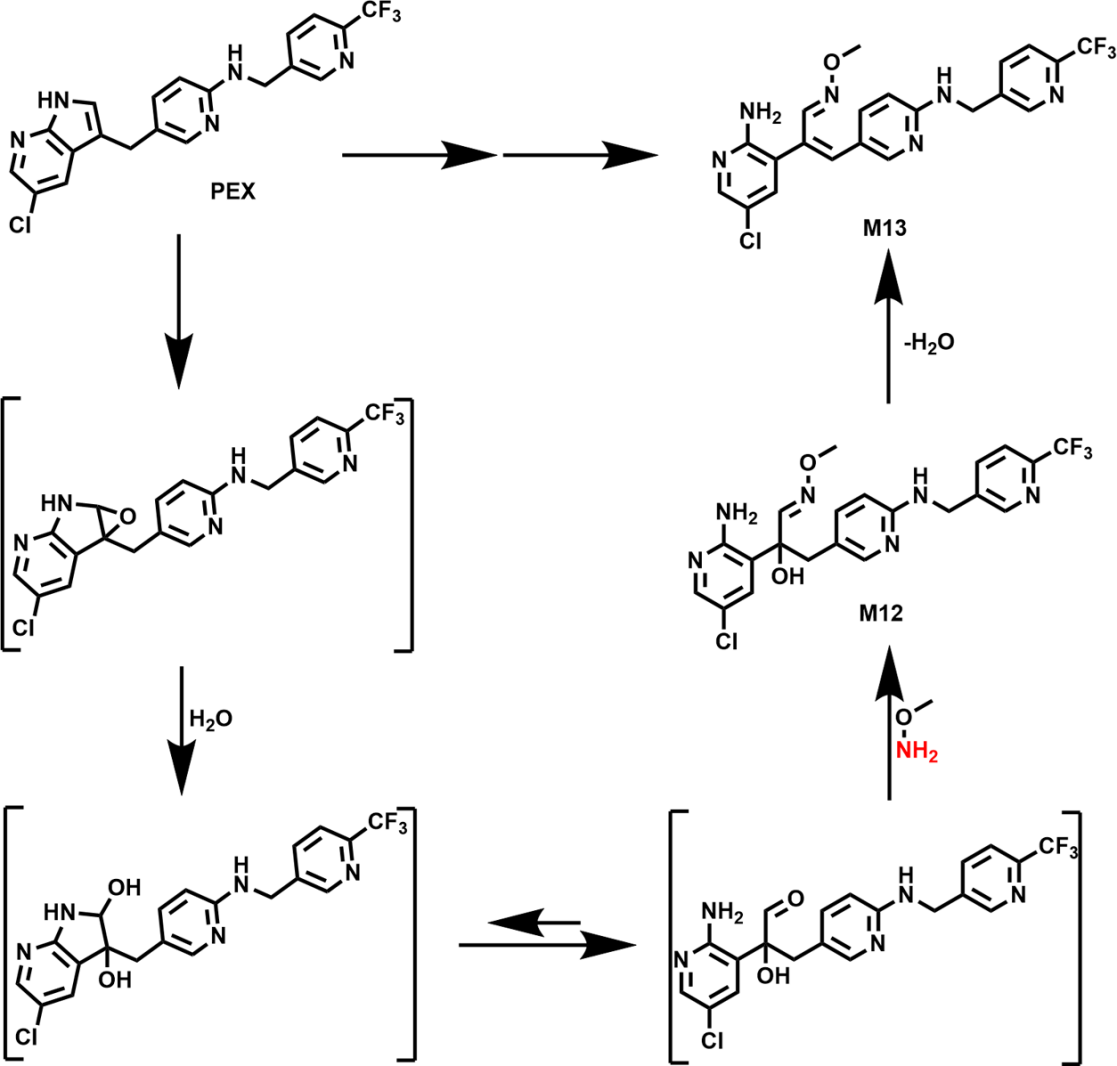
Proposed mechanism of the formation of oximes M12 and M13. M12 and M13 were detected in both HLM and MLM. The pyrrole ring of PEX was oxidized to form an epoxide, which then underwent hydrolysis to generate an aldehyde intermediate. This aldehyde intermediate reacted with NH_2_OMe to produce oxime M12. M13 was produced from M12 by eliminating H_2_O.

M12 is the dominant adduct in both HLM and MLM, followed by M13 (Figure 3B). M12 has a protonated *m*/*z* value of 481.1361 and a retention time of 9.50 min (Figure 4A). The MS/MS analysis of M12 produced fragment ions at *m*/*z* 107.0598, 160.0359, 266.0883, and 434.0971, which were elucidated in the inlaid structural diagram (Figure 4B). M13, eluted at 10.82 min, has a protonated molecule at *m*/*z* 463.1261 (Figure 4C). The MS/MS analysis of M13 produced fragment ions at *m*/*z* 257.0576, 272.0682, and 417.0940, which were elucidated in the inlaid structural diagram (Figure 4D). The pathways of M12 and M13 formation are presented in Figure 5. The pyrrole ring of PEX can be first oxidized to an epoxide. Following hydrolysis and ring opening, the aldehyde was yielded and subsequently captured by NH_2_OMe to form M12. The elimination of H_2_O from M12 produced M13 (Figure 5). M14 has a protonated *m*/*z* value of 322.1065 and is eluted at 4.74 min (Figure 4E). The MS/MS analysis of M14 generated fragment ions at *m*/*z* 107.0599, 136.0611, 257.0576, and 273.0757, which were interpreted in the inlaid structural diagram (Figure 4F). M15 eluted at 7.09 min, with a protonated molecule at *m*/*z* 304.0965 (Figure S6A and Table 1). The putative structure of M15 was elucidated based on the exact mass and predicted formula as the MS/MS data was not available (Table 1). Our previous study demonstrated that PEX could produce the amine by releasing 4-(trifluoromethyl)-benzaldehyde via dealkylation (Figure S7) in LM.^16^ As proposed in Figure S7, the formation of amine-NH_2_OMe adducts M14 and M15 followed similar pathways to the formation of M12 and M13. Oxime M17 has the protonated *m*/*z* value of 205.0583 and was eluted at 12.41 min, which was identified as the NH_2_OMe adduct of 4-(trifluoromethyl)-benzaldehyde (Figure S6D, an N-dealkylated product). The MS/MS analysis of M17 produced fragment ions at 147.0282 and 174.0390, which were elucidated in the inlaid structural diagram (Figure S6E). M16 and M18 were formed by capturing the aldehydes produced from products of carbon–carbon cleavages of PEX with NH_2_OMe (Figures S6B,C,F). Their formation pathways have been extensively studied, and their structures were confirmed with standard compounds in our previous work.^16^

### 3.5 Role of P450s in the Formation of GSH or NH2OMe Adducts Associated with PEX

**Figure 6:**
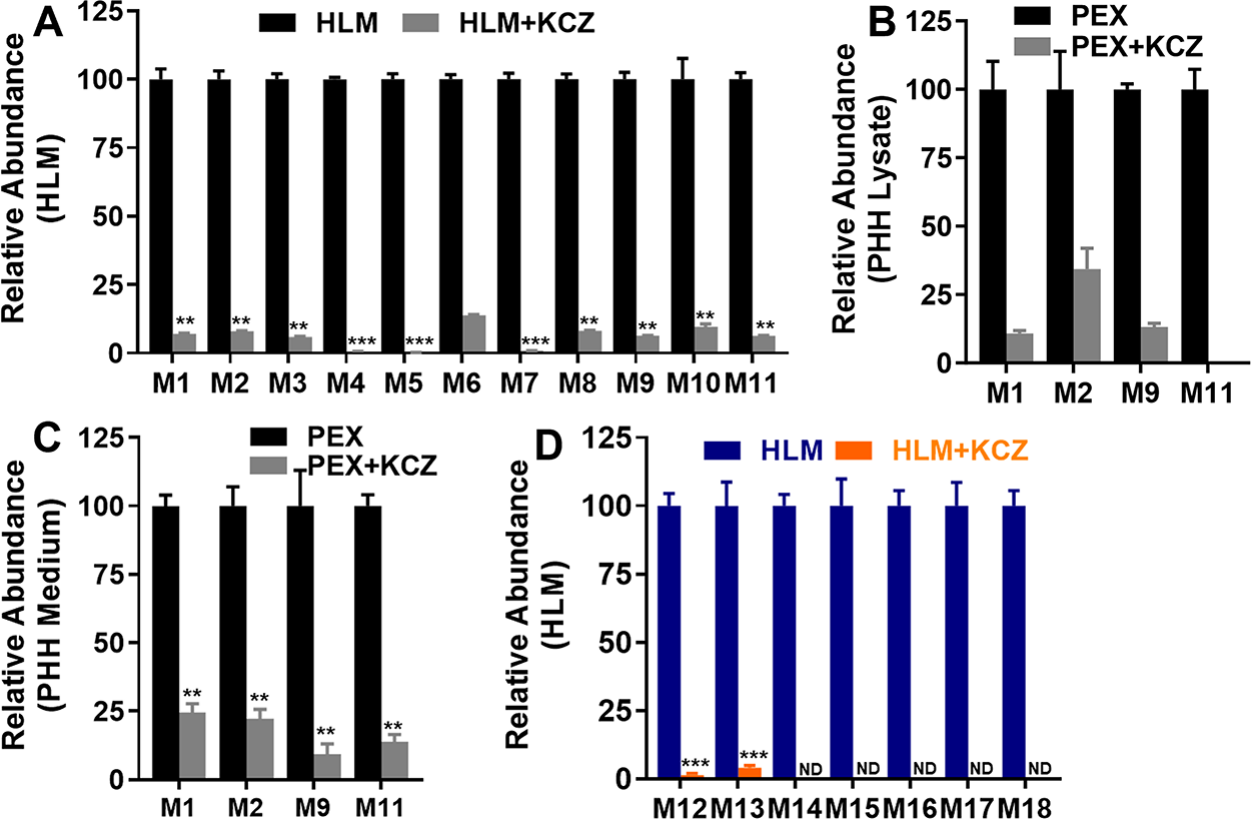
Roles of P450s in the formation of trapped reactive metabolites of PEX. KCZ, a CYP3A inhibitor, was used at a concentration of 2 μM in HLM and 10 μM in PHH for the inhibitory assays. The incubation conditions of PEX in HLM and culture condition for PHH were detailed in experimental procedures. All samples were analyzed by UHPLC-Q Exactive MS. (A) Effects of KCZ on the formations of M1–M11 in HLM. (B, C) Effects of KCZ on the formations of M1, M2, M9, and M11 in PHH lysate and medium, respectively. (D) Effects of KCZ on the formations of oximes M12–M18 in HLM. The relative abundance from the control groups without KCZ was set as 100%. All data are expressed as mean ± S.E.M (*n* = 3 for HLM; *n* =4 for PHH). Statistical analysis was conducted using a two-tailed Student′s independent *t*-test. *P < 0.05, **P < 0.01, ***P < 0.001. ND, not detected.

We investigated the role of P450s enzymes in the formation of PEX-GSH and PEX-NH_2_OMe adducts using a panel of human recombinant P450 enzymes and the specific chemical inhibitor in HLM. Using human recombinant P450 enzymes, CYP3A4 and CYP3A5 were identified as the primary enzymes responsible for the formation of adducts (M1–M15, M17, and M18). Additionally, the CYP2A6 isoenzyme contributed to the formation of M4 (31%) (Table 2). Several other isoenzymes also contributed to the formation of M16, although CYP3A4 was the major enzyme involved. In our chemical inhibitory experiments in HLM, the specific CYP3A inhibitor KCZ at a concentration of 2 μM suppressed the formation of M1-M18 by 87–100% (Figure 6A,D). In MLM, KCZ was also found to significantly inhibit the formation of the adducts, although it was less effective compared to that in HLM. The observed suppression ranged from 65 to 98% (Figures S8A,B). In PHH lysate and medium, KCZ at a concentration of 10 μM inhibited the formation of PEX-GSH adducts (M1, M2, M9, and M11), with inhibition ranging from 65 to 100% (Figure 6B,C).

**Table 2:** P450 Contribution to the Formation of PEX-GSH/PEX-NH_2_OMe Adducts^a^

	Ml	M2	M3	M4	M5	M6	M7	M8	M9	M10	Mil	M12	M13	M14	M15	M16	M17	M18
control	0.4	0.3	0.0	0.0	0.0	0.2	0.0	0.1	1.1	0.0	0.0	0.4	0.7	0.0	0.0	7.5	1.3	0.0
CYP1A2	1.4	1.2	0.3	0.0	0.0	1.0	0.0	0.4	2.1	9.7	0.0	1.1	1.3	0.0	0.0	10.4	3.6	6.4
CYP2A6	2.2	2.4	0.6	31.3	0.0	0.2	0.0	0.3	1.0	0.0	0.0	0.5	1.8	0.0	0.0	30.3	3.2	0.0
CYP2B6	0.7	0.4	0.1	0.0	0.0	0.2	0.0	3.7	1.9	0.0	0.0	0.4	0.9	0.0	0.0	9.0	2.4	8.5
CYP2C8	0.4	0.4	0.1	0.0	0.0	0.3	0.0	0.9	1.1	0.0	0.0	0.7	0.8	0.0	0.0	11.6	2.4	2.9
CYP2C9	0.4	0.3	0.1	0.0	0.0	0.2	0.0	0.1	0.9	0.0	0.0	0.4	0.7	0.0	0.0	4.5	1.5	9.6
CYP2C19	1.0	0.6	0.1	0.0	0.0	0.4	0.0	0.5	1.2	0.0	0.0	0.6	1.0	0.0	0.0	14.2	2.3	4.8
CYP2D6	6.9	7.1	2.2	10.4	0.0	12.6	0.0	5.3	5.2	2.2	0.0	13.3	9.7	0.0	0.0	48.9	6.0	0.0
CYP2E1	0.6	0.4	0.0	0.0	0.0	0.3	0.0	0.2	0.7	0.0	0.0	0.5	0.6	0.0	0.0	2.7	2.5	5.1
CYP3A4	100.0	100.0	100.0	100.0	96.4	100.0	95.8	100.0	100.0	93.8	100.0	100.0	100.0	63.5	100.0	100.0	52.7	100.0
CYP3A5	51.4	46.4	54.4	56.3	100.0	62.2	100.0	71.0	38.7	100.0	33.4	66.3	50.0	100.0	90.3	46.5	100.0	22.1

a cDNA-expressed human P450 enzymes (control, CYP1A2, 2A6, 2B6, 2C8, 2C9, 2C19, 2D6, 2E1, 3A4, and 3A5) were used to determine the role of individual P450 isoforms in PEX metabolism. All samples were analyzed by UHPLC-Q Exactive MS. The largest peak area of the individual metabolite produced by P450 isoforms was set as 100%. GSH, glutathione; NH_2_OMe, methoxyamine.

## 4 Discussion

Metabolomics-based strategies have been widely adopted for studying both stable and reactive drug metabolites.^17−20,22^ Reactive drug metabolites are well appreciated as significant contributors to drug adverse effects due to their potential for covalent modification of important biological macromolecules such as proteins and DNA.^14,17^ Comprehensive understanding of the metabolic profile and characterizing reactive metabolites of drug benefit drug safety evaluation and improvement. In our previous work, we identified stable phase I metabolites of PEX, indicating extensive CYP3A-mediated metabolism of PEX in vitro.^16^ In this study, we employed an LC–MS-based metabolomic approach to screen for trapped reactive metabolites related to PEX. A total of 11 PEX-GSH and 7 PEX-NH_2_OMe adducts were identified in HLM or MLM incubations. The formation of GSH adducts associated with PEX suggests the presence of active metabolites during in vitro metabolism, indicating the possibility of a similar reaction occurring between PEX and free sulfur groups in proteins, which could impair the functions of important macromolecules in vivo. In PHH lysate and medium, M1 and M2 were identified, showing a similar pattern to those in HLM/MLM, while the major adduct M6 observed in HLM/MLM was not detected in PHH (Figure 1C,D). It is possible that certain enzymes in PHH directly convert M6 to M1 or M2 by eliminating H_2_O, resulting in the absence of M6 in PHH. Furthermore, the compositions of PEX-GSH adducts differ between PHH lysate and culture medium (Figure 1D). M9 (Cleave-left-GSH adduct) has the largest peak area in PHH lysate, while it is smaller in PHH medium. Further investigation is necessary to establish the extent of the involvement of GSH-trapped reactive metabolites in PEX toxicity, as their roles are currently not well understood.

The NH_2_OMe is commonly used in vitro for trapping aldehydes in vitro, as they are often considered toxic metabolites.^17,19,23^ By employing metabolomic strategies in conjunction with manual extraction to profile the PEX-NH_2_OMe adducts, we identified 7 oximes, indicating the formation of active aldehyde metabolites (Figure 3A,B, Table 1). Previous studies have suggested that the interaction between aldehydes and the exocyclic amino groups of DNA and the ε-amino groups of lysine residues can lead to the formation of cross-links between deoxynucleotides and protein amino acids.^24,25^ These interactions may cause toxicity by impairing the function of macromolecules.^26^ Additional research is required to thoroughly examine the contribution of aldehyde in PEX toxicity and better understand its role.

**Figure 7:**
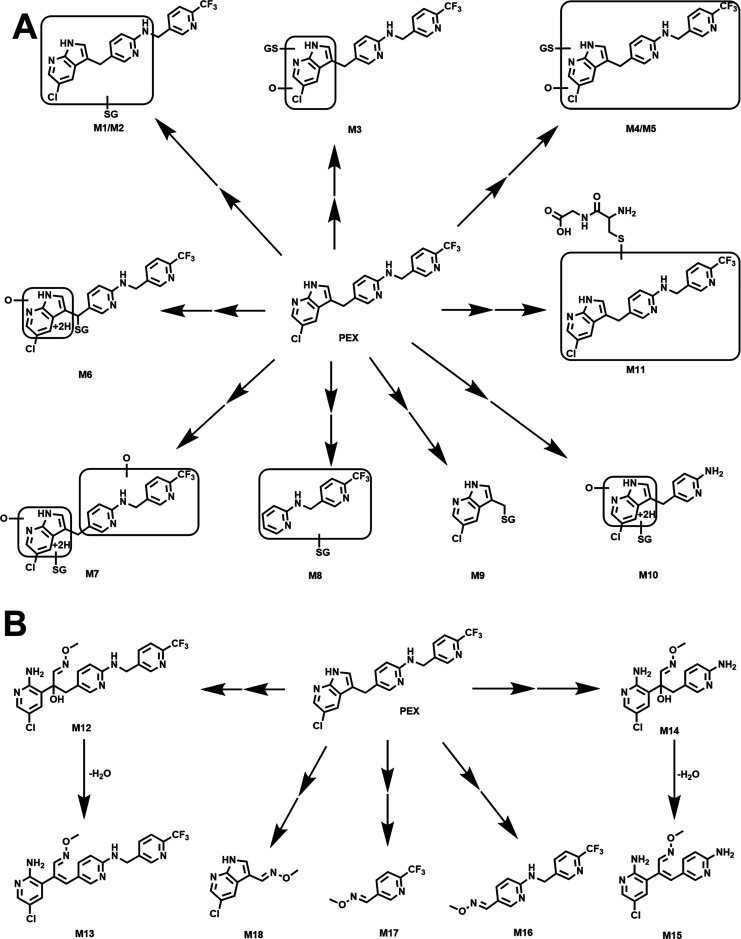
Summary of trapped PEX reactive metabolites. The structures were determined based on the exact mass (mass error less than 5 ppm) and MS/MS fragments. The putative structures of M9–M11 and M15 were determined based on the exact mass and predicted formulas. (A) Metabolic map of PEX-GSH adducts. (B) Metabolic map of PEX-NH_2_OMe adducts.

The GSH adducts identified in both HLM and MLM were M1, M2, M6, and M9 (Figure 1C). Species differences were observed in the formation of M1, M2, and M9 between HLM and MLM. HLM produced more M1 and M9 than MLM, while the percentage of M2 was higher in MLM than in HLM. As shown in Figure 2B,C, M1 and M2 represented PEX-GSH adducts by conjugating one molecule of GSH to the different sites on PEX. M3–M7 were formed by conjugating GSH to oxidized PEX metabolites, which were observed in PEX metabolism in HLM and MLM.^16^ M11 (PEX-Cys-Gly) was likely produced from M1 or M2 by the loss of the glutamic acid motif. The exact mechanisms of formation for M1, M2, and M11 remain unclear. Adducts M8–M10 are three GSH adducts formed by conjugating GSH with cleaved parts of PEX (Figures S1 and S4). Previous studies suggest that PEX can form phenol metabolites through carbon–carbon cleavage.^16^ We suspected that the phenol metabolite could react with GSH, followed by the loss of H_2_O to form M8; however, incubation of the phenol metabolite with GSH in HLM did not yield M8, indicating that M8 formation from PEX occurred via alternative unknown pathways. We have determined the formation of M9 in the study of CYP3A-mediated carbon–carbon cleavage of PEX.^16^ In brief, the formed cation conjugates with GSH to form M9 (Figure S1 and Figure 7). PEX can undergo dealkylation to form the amine metabolite (Figure S7). Following further oxidization and hydrogenation, the resulting metabolite can be modified by GSH to form M10 (Figure 7). Unfortunately, for some minor metabolites like M4, M10, and M11 (Figure S4), the MS/MS spectra of high quality were not obtained due to their low abundance. The tentative structures were speculated based on their exact masses.

The formation of the oximes (M12–M18) in HLM and MLM indicated the generation of reactive aldehydes during PEX metabolism. The formation of M12 and M13 is proposed in Figure 5. The process involves the oxidation of the pyrrole ring of PEX to form an epoxide, which then undergoes hydrolysis to form an aldehyde intermediate. This aldehyde intermediate then reacts with NH_2_OMe to produce oxime M12. M13 can be formed from M12 by eliminating H_2_O. An alternative pathway involves the aldehyde losing H_2_O first and subsequently reacting with NH_2_OMe to produce M13. As shown in Figure S7, the formation of M14 and M15 followed a similar process when the amine served as a substrate. Among the oximes M16–M18, the formation of M16 has been detailed in our carbon–carbon cleavage paper.^16^ The aldehyde M16 itself has also been detected in both HLM and MLM (Figure S7). M17 is the product of the condensation of 4-(trifluoromethyl)benzaldehyde with NH_2_OMe. The formation of benzaldehyde in PEX metabolism was further confirmed by the detection of its corresponding acid and alcohol. The structures of these compounds were also validated using their standard compounds.

Among the tested P450 isoforms, CYP3A4 and CYP3A5 were identified as the primary enzymes responsible for the formation of the stable metabolites of PEX in HLM and the adducts of its reactive metabolites^16^ (Table 2 and Figure 6A). Our findings are supported by the clinical data that inhibiting PEX metabolism should lead to higher PEX concentrations in vivo. In clinical practice, the potent CYP3A inhibitor itraconazole significantly increases PEX exposure in healthy human subjects.^27^ It was observed that the oxime M16 can be produced by various recombinant human P450s, and even in the control group. Nevertheless, CYP3A enzymes remain the primary contributors as shown in Table 2, as the formation of M16 in HLM and MLM was significantly inhibited by the CYP3A inhibitor KCZ, with inhibition levels reaching up to 100% (Figure 5A,B). These findings suggest that CYP3A isoforms are the primary enzymes involved in M16 formation in HLM, while some unknown components in the recombinant human P450s may also produce the aldehyde. Additional studies are required to investigate the roles of CYP3A-mediated metabolism and reactive metabolites in PEX hepatotoxicity.

In summary, we conducted a comprehensive study on the reactive metabolites of PEX in HLM, MLM, and PHH using an LC–MS-based metabolomic approach and trapping agents (GSH and NH_2_OMe). A total of 11 GSH adducts and 7 NH_2_OMe adducts associated with PEX were identified (Figure 7). Among these, 4 GSH adducts were also detected in PHH. Among the tested P450 isoforms, CYP3A4 and CYP3A5 were identified as the primary enzymes contributing to the formation of these adducts. The formation of these adducts suggested that PEX metabolism could produce reactive metabolites, which have the potential of modifying important biological macromolecules. Further studies are warranted to elucidate the potential association of these reactive metabolites with PEX-related adverse effects, especially liver injury.

## Supporting Information Available

Proposed pathways of the formation of M9; trend plots of M1 and M8 generated by OPLS-DA analysis; MS/MS of PEX; identification of GSH adducted related to PEX M10 and M11; metabolomic screening of the PEX-NH_2_OMe adducts in human liver microsomes; identifying oximes M15–M18; proposed mechanism of the formation of oximes M14–M18; and roles of mouse Cyp3a in the formation of trapped reactive metabolites of PEX (PDF)

## Abbreviations

PEX: pexidartinib
P450: cytochrome P450
GSH: reduced glutathione
NH_2_OMe: methoxyamine
HLM/MLM: human/mouse liver microsomes
PHH: primary human hepatocytes
KCZ: ketoconazole
NADPH: reduced β-nicotinamide adenine dinucleotide 2′-phosphate
PBS: phosphate-buffered saline
OPLS-DA: orthogonal projection to latent structures-discriminant analysis
UHPLC: ultrahigh-performance liquid chromatography
Q Exactive MS: Q Exactive Hybrid Quadrupole-Orbitrap Mass Spectrometer
Cys: cysteine
Gly: glycine
