# Supplementary material for: Identifying the Reactive Metabolites of Tyrosine Kinase Inhibitor Pexidartinib In Vitro Using LC–MS-Based Metabolomic Approaches
Source: Chem Res Toxicol. 2023 Aug 2;36(8):1427–38. doi: 10.1021/acs.chemrestox.3c00164 (PMC10445284; doi:10.1021/acs.chemrestox.3c00164)

Supporting Information

Identifying the reactive metabolites of tyrosine-kinase inhibitor pexidartinib in vitro using LC-MS-based metabolomic approaches

Xuan Qin[1], Yong Wang[1], Kevin R. MacKenzie[1,2,3], John M. Hakenjos[1], Si Chen[4], Saleh M. Khalil[1], Sung Yun Jung[5], Damian W. Young[1,3], Lei Guo[4], and Feng Li[1,2,3]*

[1]Center for Drug Discovery, Department of Pathology & Immunology, Baylor College of Medicine, Houston, TX 77030, USA

[2]NMR and Drug Metabolism Core, Advanced Technology Cores, Baylor College of Medicine, Houston, TX 77030, USA

[3]Department of Pharmacology & Chemical Biology, Baylor College of Medicine, Houston, TX 77030, USA

[4]Division of Biochemical Toxicology, National Center for Toxicological Research/U.S. Food and Drug Administration (FDA), Jefferson, AR 72079, USA

[5]Department of Molecular & Cellular Biology, Baylor College of Medicine, Houston, TX 77030, USA

Correspondence: Feng Li, PhD

Center for Drug Discovery,

Department of Pathology & Immunology,

Baylor College of Medicine,

Houston, TX 77030

Tel: 001-713-798-3623

Email: fl3@bcm.edu

Table of Contents

Figure S1. Proposed pathways of the formation of M9. S3

Figure S2. Trend plots of M1 and M8 generated by OPLS-DA analysis. S4

Figure S3. MS/MS of PEX. S5

Figure S4. Identification of GSH adducted related to PEX M10 and M11. S5

Figure S5. Metabolomic screening the PEX-NH_2OMe adducts in human liver microsomes. S6

Figure S6. Identifying oximes M15-M18. S7

Figure S7. Proposed mechanism of the formation of oximes M14-18. S8

Figure S8. Roles of mouse Cyp3a in the formation of trapped reactive metabolites of PEX. S9

Figure S1. Proposed pathways of the formation of M9.

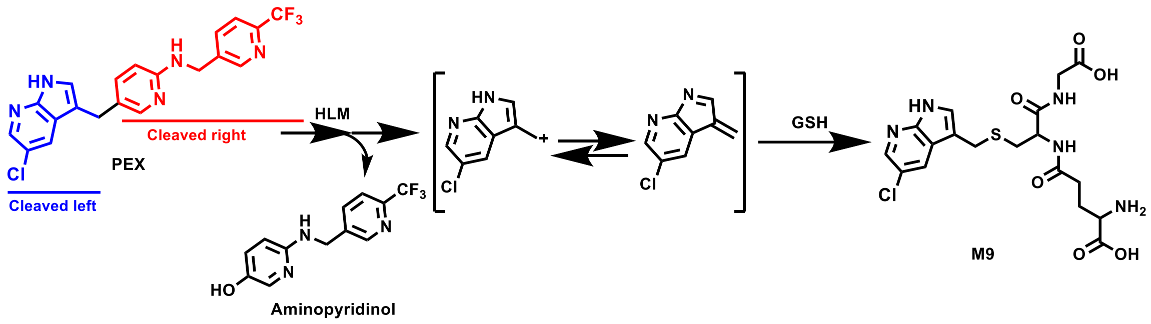

Figure S2. Trend plots of M1 and M8 generated by OPLS-DA analysis.
Metabolomic analysis of control groups (HLM+PEX+NAPDH and HLM+PEX+GSH-NAPDH) and PEX group (HLM+PEX+GSH+NAPDH) was conducted. The trend plot was generated from OPLS-DA analysis. (A)Trend plot of M1. (B) trend plot of M8.

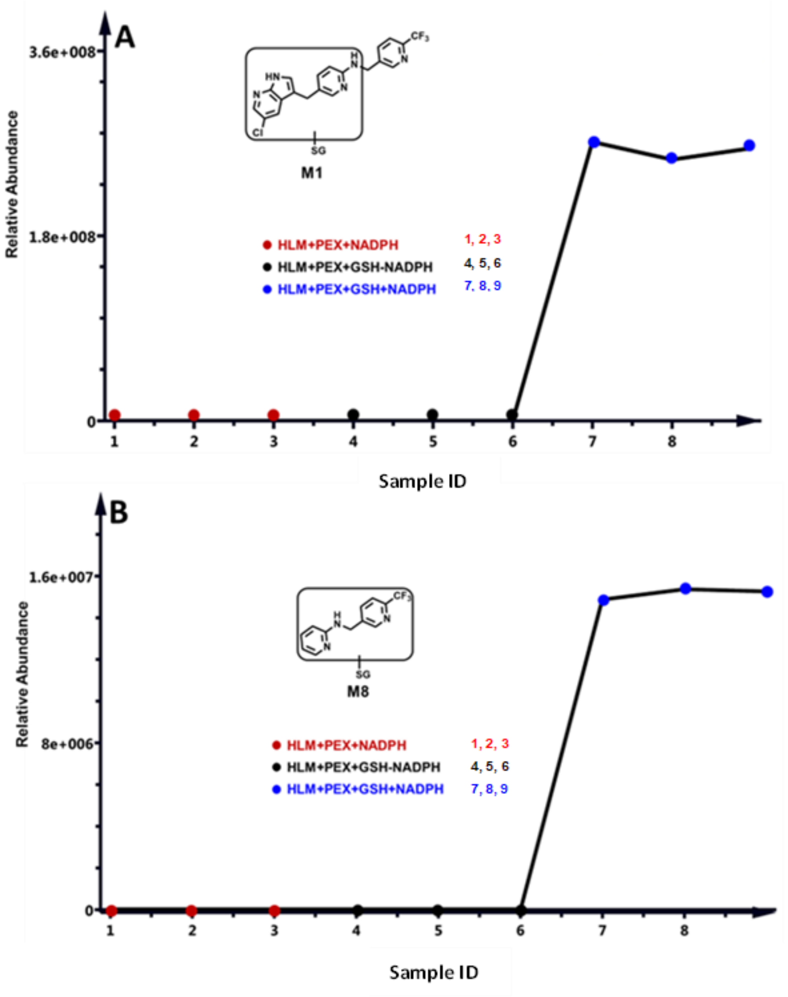

Figure S3. MS/MS of PEX.

MS/MS was performed with collision energy ramping from 10–35 eV. The major fragmental ions are interpreted in the insets.

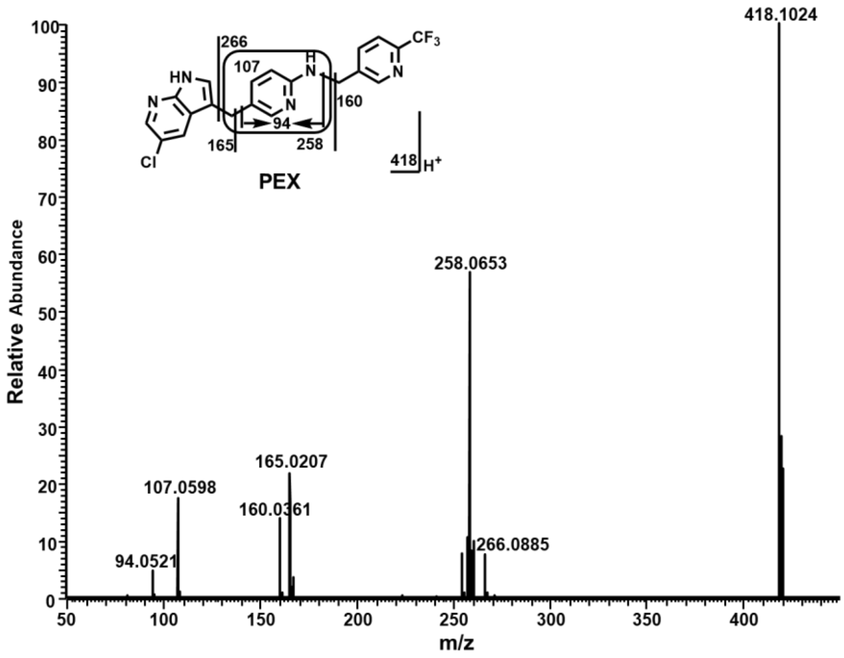

Figure S4. Identification of GSH adducted related to PEX M10 and M11.

Incubations were conducted in 1× phosphate-buffered saline (1× PBS, pH 7.4), containing 30 μM PEX, 0.1 mg LM, 2.5 mM GSH, and NADPH (final concentration 1.0 mM) in a final volume of 100 μL. All the samples were analyzed using UHPLC-Q Exactive MS. (A) Chromatogram of M10. (B) Chromatogram of M11.

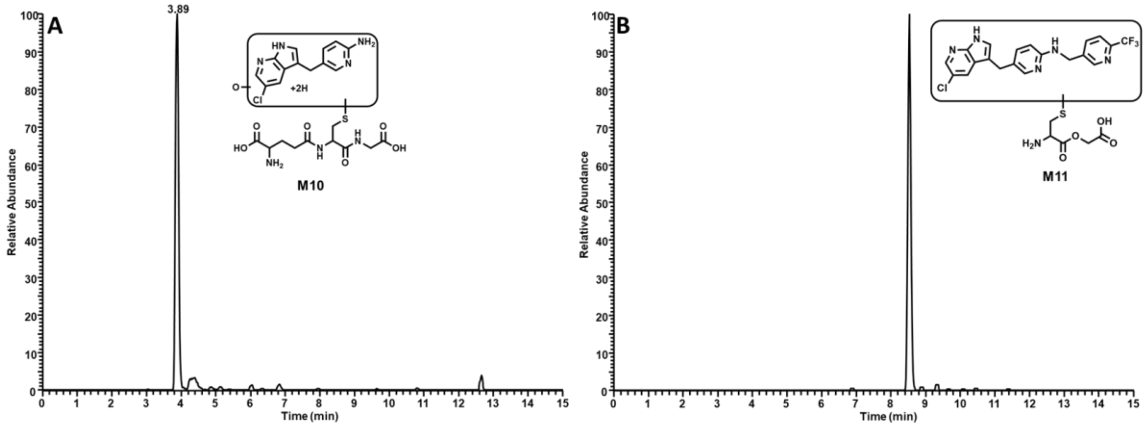

Figure S5. Metabolomic screening the PEX-NH_2OMe adducts in human liver microsomes. Metabolomic analysis of control groups (HLM+PEX+NAPDH and HLM+PEX+ NH_2OMe-NAPDH) and PEX group (HLM+PEX+ NH_2OMe+NAPDH) was conducted. (A) Separation of control and PEX group in OPLS-DA score plot. The t[1] and to[1] values represent the score of each sample in principal component 1 and 2, respectively. (B) Trend plot of M12 generated by OPLS-DA analysis.

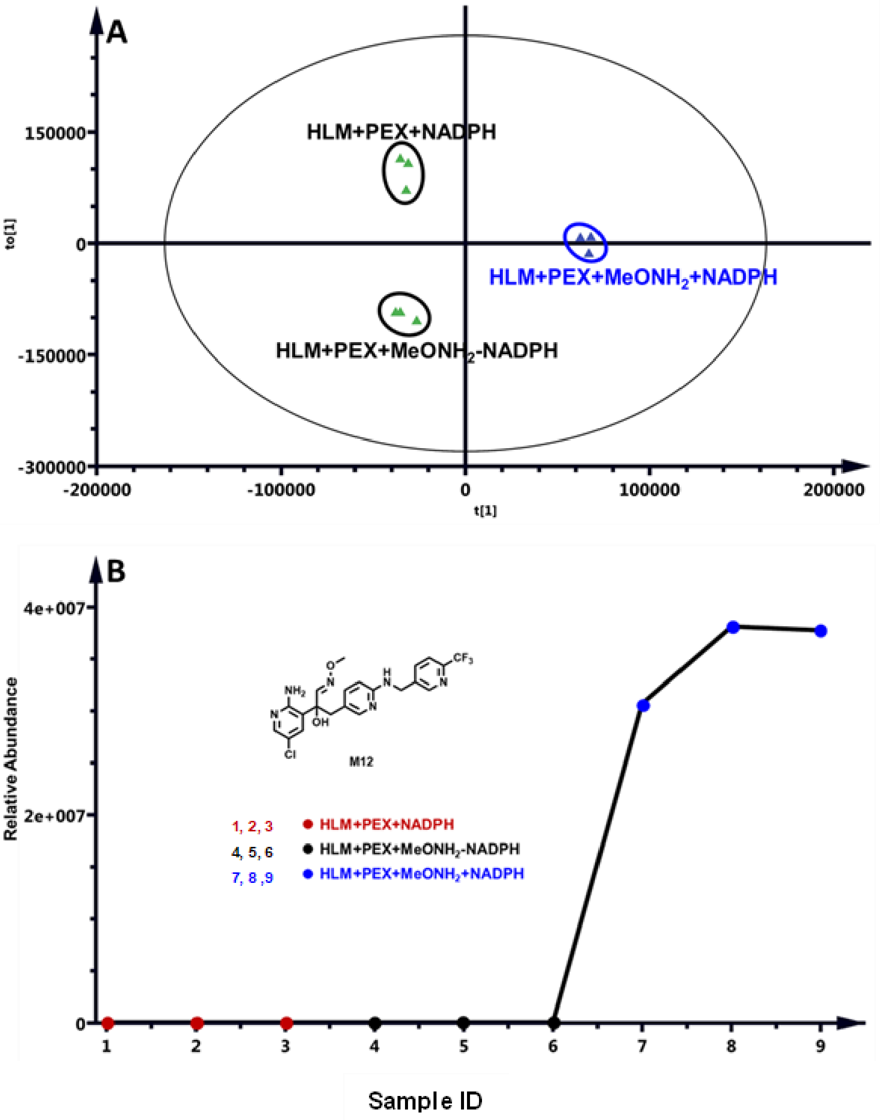

Figure S6. Identifying oximes M15-M18.

Incubations were conducted in 1× phosphate-buffered saline (1× PBS, pH 7.4), containing 30 µM PEX, 0.1 mg LM, 2.5 mM NH_2OMe, and NADPH (final concentration 1.0 mM) in a final volume of 100 µL. All the samples were analyzed using UHPLC-Q Exactive MS. MS/MS was performed with the normalized collision energy ranging from 10-35 eV. (A) Chromatogram of M15. (B) Chromatogram of M16. (C) MS/MS of M16. (D) Chromatogram of M17. (E) MS/MS of M17. (F) Chromatogram of M18. (G) MS/MS of M18.

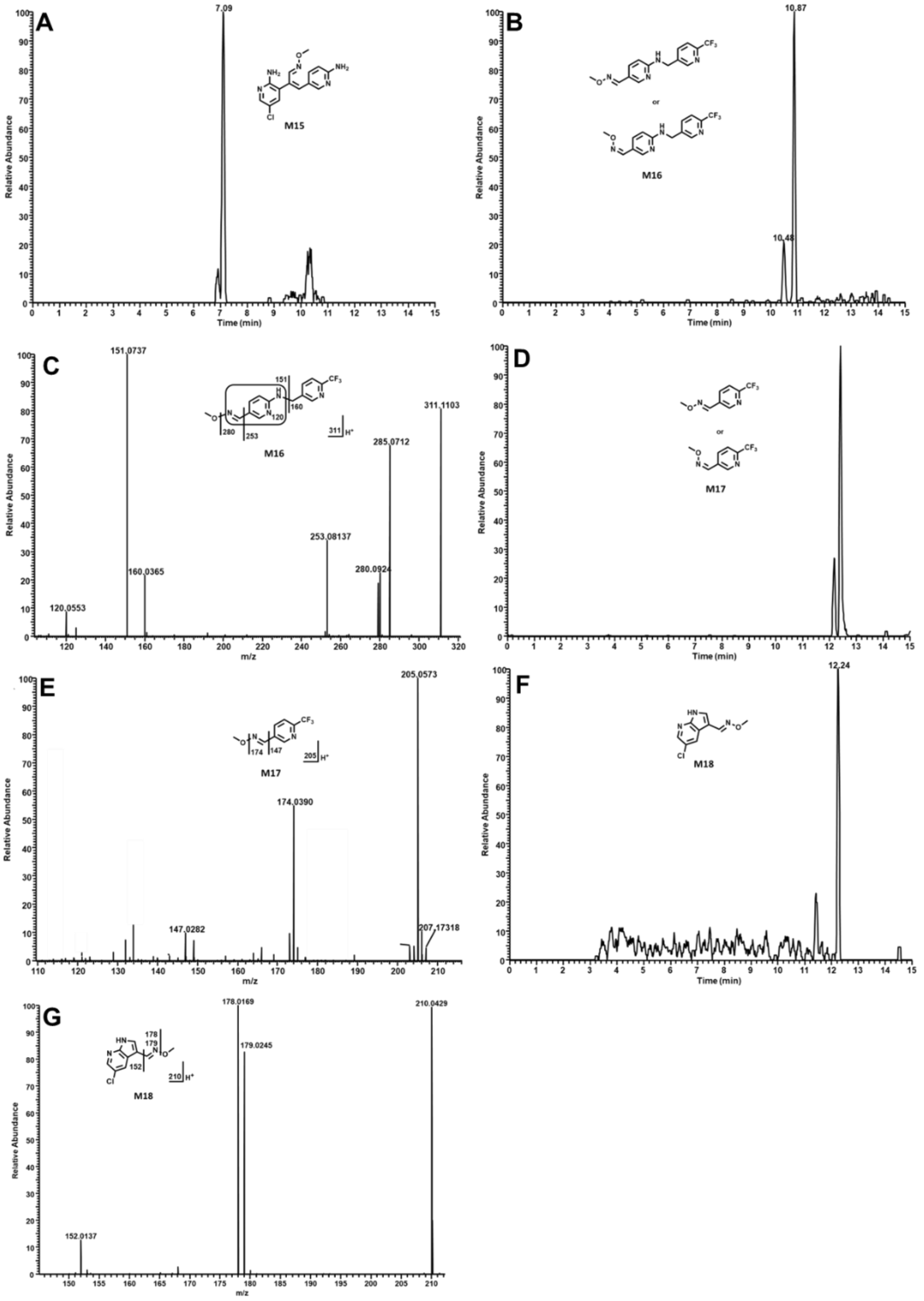

Figure S7. Proposed mechanism of the formation of oximes M14-18.

M14-M18 were detected in HLM and MLM. PEX produced the amine by releasing the 6-CF_3-nicotinaldehyde via N-dealkylation. The aldehyde reacted with NH_2OMe to form oxime M17. The pyrrole ring of amine was oxidized to form an epoxide, which then underwent hydrolysis to generate an aldehyde intermediate. This aldehyde intermediate reacts with NH_2OMe to produce oxime M14. M15 was produced from M14 by eliminating H_2O. Carbon-carbon bond cleavage yield nicotinaldehyde and pyridinylmethanol. The nicotinaldehyde was trapped with NH_2OMe to form oxime M16. The pyridinylmethanol was further oxidized to generate the corresponding aldehyde, which was captured by NH_2OMe to form M18.

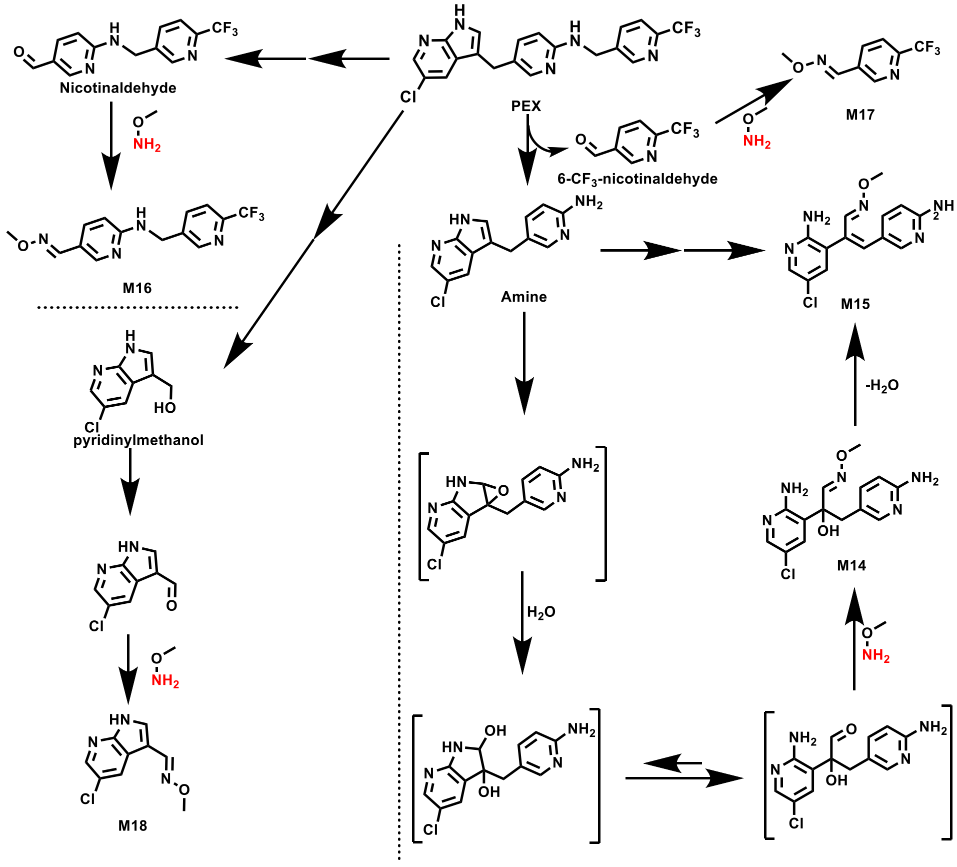

Figure S8. Roles of mouse Cyp3a in the formation of trapped reactive metabolites of PEX. KCZ (CYP3A inhibitor, 2 μM) in MLM was used in the inhibitory assays. The incubation conditions of PEX in MLM were detailed in experimental procedures. All samples were analyzed by UHPLC-Q Exactive MS. (A) Effects of KCZ on the formations of M1-M11 in MLM. (B) Effects of KCZ on the formations of oximes M12-M18 in MLM. The relative abundance from the control groups without KCZ was set as 100%. All data are expressed as mean ± S.E.M (n = 3). Statistical analysis was conducted using two-tailed Student's independent *t*-test. $^{**}P<0.01$, $^{***}P<0.001$.

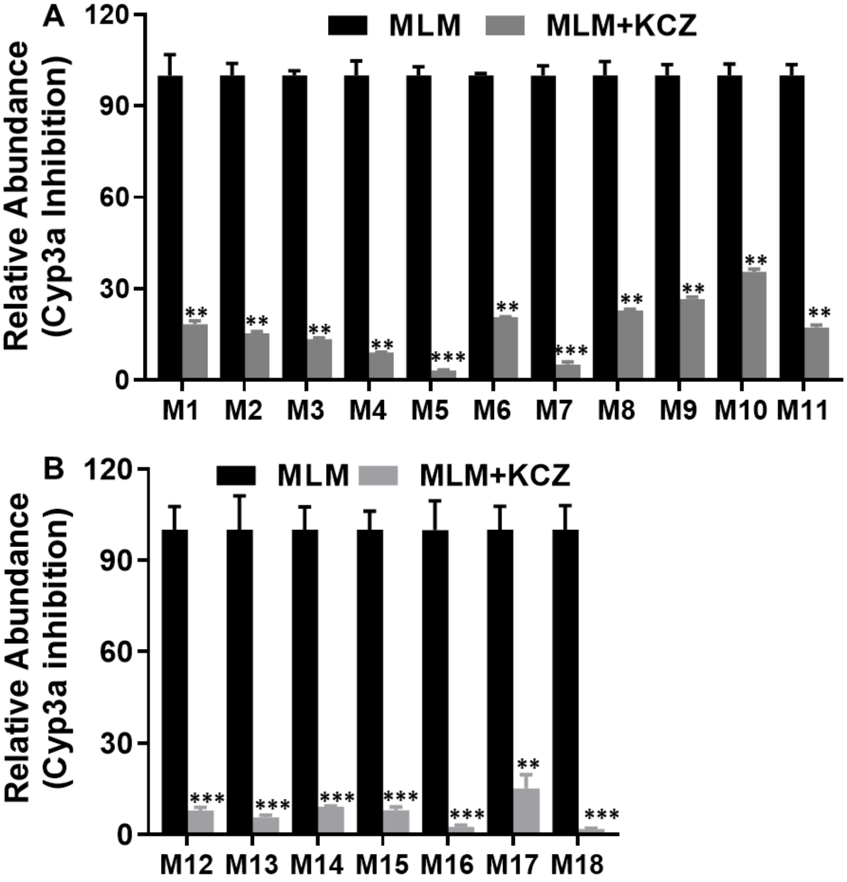

Supplement: Supplementary file 1 — tx3c00164_si_001.pdf [file tx3c00164_si_001.pdf]
